# A comparison of propofol-to-BIS post-operative intensive care sedation by means of target controlled infusion, Bayesian-based and predictive control methods: an observational, open-label pilot study

**DOI:** 10.1007/s10877-018-0208-2

**Published:** 2018-10-11

**Authors:** M. Neckebroek, C. M. Ionescu, K. van Amsterdam, T. De Smet, P. De Baets, J. Decruyenaere, R. De Keyser, M. M. R. F. Struys

**Affiliations:** 1 Department of Anesthesia and Perioperative Medicine, Ghent University Hospital, 9000 Ghent, Belgium; 2 Research Group on Dynamical Systems and Control (DySC), Department of Electrical Energy, Mechanical Constructions and Systems, Ghent University, Metals, Ghent, Belgium; 3 Department of Anesthesiology, University Medical Center Groningen, University of Groningen, Hanzeplein 1, 9700 RB Groningen, The Netherlands; 4 Demed Medical, Temse, Belgium; 5 Department of Intensive Care Medicine, Ghent University, Ghent, Belgium; 6 Department of Anesthesia and Perioperative Medicine, Ghent University, Ghent, Belgium

**Keywords:** Propofol, Bispectral index, Closed-loop, Intensive care sedation

## Abstract

**Purpose:**

We evaluated the feasibility and robustness of three methods for propofol-to-bispectral index (BIS) post-operative intensive care sedation, a manually-adapted target controlled infusion protocol (HUMAN), a computer-controlled predictive control strategy (EPSAC) and a computer-controlled Bayesian rule-based optimized control strategy (BAYES).

**Methods:**

Thirty-six patients undergoing short lasting sedation following cardiac surgery were included to receive propofol to maintain a BIS between 40 and 60. Robustness of control for all groups was analysed using prediction error and spectrographic analysis.

**Results:**

Although similar time courses of measured BIS were obtained in all groups, a higher median propofol effect-site concentration (CePROP) was required in the HUMAN group compared to the BAYES and EPSAC groups. The time course analysis of the remifentanil effect-site concentration (CeREMI) revealed a significant increase in CeREMI in the EPSAC group compared to BAYES and HUMAN during the case. Although similar bias and divergence in control was found in all groups, larger control inaccuracy was observed in HUMAN versus EPSAC and BAYES. Spectrographic analysis of the system behavior shows that BAYES covers the largest spectrum of frequencies, followed by EPSAC and HUMAN.

**Conclusions:**

Both computer-based control systems are feasible to be used during ICU sedation with overall tighter control than HUMAN and even with lower required CePROP. EPSAC control required higher CeREMI than BAYES or HUMAN to maintain stable control.

Clinical trial number: NCT00735631.

**Electronic supplementary material:**

The online version of this article (10.1007/s10877-018-0208-2) contains supplementary material, which is available to authorized users.

## Introduction

Short lasting sedation following cardiac surgery has become an integral part of post-operative intensive care [1–4]. Clinical experience has revealed that standard dosing guidelines often results in an inaccurate level of sedation due to a wide range of inter-patient pharmacological variability. A thorough patient-individualized approach is required and independent depth of anesthesia (DOA) systems based on the processed EEG such as the Bispectral Index (BIS®, Medtronic, Dublin, Ireland) might assist in optimizing sedation at the intensive care unit (ICU) [5–7].

Clinicians select initial drug regimen on the basis of a variety of considerations and adjust where needed. In control engineering terminology, this series of action constitutes a closed-loop control system. Human-controlled closed-loop is characterized by irregular control actions which are intermittent in time. To solve this, computer-controlled closed-loop applications have been developed to control drug administration during intra-operative anesthesia. And several single input single output (SISO) control algorithms have been tested [8, 9]. The earliest anesthesia controllers use three-term controllers such as proportional integral derivative (PID) [10].The more recent employs sophisticated modelling [6, 11–13]. Although PID controllers are popular tools from control engineering areas [14, 15], they do not capture the true activity in the clinic. PID controllers cannot anticipate to the response of the patient, so stability might become a problem [16]. Strategies, such as fuzzy [17, 18], adaptive [19], predictive [20–22] and Bayesian based closed-loop [23–26] control algorithms have been suggested.

It has been shown that significant time delays are induced from signal processing algorithms and these become important for the stability of the closed loop [27]. Some uncertainties may come from the patient model as well, and adaptations have been suggested [28]. The variability may introduce variations as high as threefold [29].

Closed-loop delivery of propofol during intra-operative anesthesia is found to be superior to manually controlled delivery [9], however, closed-loop sedation using BIS-guided propofol administration in the ICU is not well-studied. Only one feasibility study using a PID-based control strategy showed that closed-loop delivery of propofol to control BIS for postoperative sedation is feasible and efficient after cardiac surgery [30]. No studies are available comparing various automated drug delivery systems using different control algorithms.

The aim of this observational, open-label pilot study was to observe the feasibility and robustness of various approaches for single-input-single-output closed-loop control of BIS-guided postoperative propofol sedation at the ICU. The primary outcome measures were the performance of the control system to maintain a specific BIS target and the required propofol and remifentanil predicted effect-site concentrations, defined as CePROP and CeREMI, and dosages to do so. The secondary outcome measure was the hemodynamic stability of the patient. Three closed-loop control settings were tested, i.e. the predictive control (“EPSAC”), the Bayesian-based control (“BAYES”), and the routine clinical infusion (“HUMAN”).

## Methods

### Clinical protocol

This study is an observational, open-label pilot study using one computer system (RUGLOOP II, Demed, Temse Belgium) with multiple settings, being Bayesian, EPSAC and human closed-loop control. No blinded randomization was done. The inclusion was done in a blocked way, using consecutive patients entering the ICU.

After receiving Ethics’ Committee approval (University Hospital, Ghent, Belgium), patients’ informed consent and registration at ClinicalTrials.gov (NCT00735631), patients entering the ICU following off-pump coronary artery bypass surgery (OPCAB) older than 18 years were included between June 2008 and September 2009. Exclusion criteria were patients with severe renal failure defined by the RIFLE Classification levels risk, failure and end-stage kidney failure, severe hepatic failure defined by a bilirubin level of ≥ 3 mg/dl and/or a prothrombin level of < 50%, low ejection fraction defined as < 40%, age < 18 years, postoperative bleeding exceeding 2 ml/kg/hr within the first 2 h postoperatively and exceeding 1 ml/kg/hr in the following hours, history of cerebrovascular accident, history of COPD, age > 75 years, postoperative cardiac index < 2.5 for more than 2 h, SvO_2_ < 60% for more than 2 h, hypotension with a MAP < 60 mmHg for more than 2 h, sedation agents other than remifentanil and propofol during surgery or remifentanil dose exceeding 0.5 µg/kg/min at arrival at the ICU.

All patients have been routinely monitored. Patients had been admitted to the ICU while sedated. At the ICU, patients were sedated with propofol using one of the three SISO closed-loop systems. For supplemental analgesia control, remifentanil target-controlled infusion (TCI) was given. CePROP and CeREMI were calculated using the Schnider and Minto pharmacokinetic models, respectively [31–34].

The SISO systems guided the sedation maintaining BIS targets between 40 and 60 aiming to be as close as possible around 50. Additionally, all vital signs were recorded by RUGLOOP II data collecting software (Demed, Temse, Belgium).

### Patient models for prediction

In order to control the depth of sedation, a model which captures the dynamical response of the patient is required. The selection of the model variables is crucial. The most commonly used drugs are propofol and remifentanil.

Propofol is a hypnotic agent, well described and studied [31, 32] and used as a sedative agent in ICU. Remifentanil is an opioid with a unique pharmacologic profile, characterized by its high metabolic clearance [33, 34], used as a strong analgesic. When administered together, these two drugs interact synergistically. These two drugs are the inputs of the model and the output is the BIS, a signal derived from the electroencephalogram (EEG), used as the measure of the hypnotic component of anesthesia. Multivariate statistics were used to combine the different features into a single indicator value. BIS values lie in the range from 0 to 100: 100 being awake and 60 to 40 indicate light and moderate hypnotic state, respectively [35].

In SISO regulatory loops of propofol, the concentration–response relation, i.e. the pharmacodynamic (PD) model, can be described by:

1 $$BIS(t)={E_0} - {E_{\hbox{max} }}\frac{{{C_e}{{(t)}^\gamma }}}{{{C_e}{{(t)}^\gamma }+C_{{50}}^{\gamma }}}$$

where *γ* is the slope of the PD model; *E*_*max*_ is the maximum possible drug effect, with the predicted effect-site concentration *C*_*e*_(*t*). Since the parameters of (1) are unknown and different for each patient, nominal values have been used for the simulations. The nominal PD values were C_50_ = 2.5 µg/ml and γ = 3.01. E_max_ and E_0_ have been considered equal to the value of 100.

The BAYES closed loop system adapts the parameters of (1) continuously to the real BIS values and infused propofol rate profiles, which makes the BAYES controller patient-specific. The EPSAC controller uses these nominal values, requiring an increased robustness to patient model uncertainties.

### Control systems

The SISO control systems were embedded into the RUGLOOP II computer system, which stores all vital signs data collected from the various monitors using RS 232 interfaces and contains the required TCI software to steer two infusion pumps (Carefusion, Asena GH, Basingstoke, UK). The technology of TCI has been published elsewhere [36]. In a TCI system, a desired (“target”) drug concentration is set, and adjusted based on clinical observation of the response of the patient. In this study, the required target CeREMI can be set at the computer screen when using the “human” SISO controller or are set by the closed-loop system when using the Bayesian or EPSAC closed-loop system.

For the Bayesian SISO controller (called *BAYES*), an automated system was used that had previously undergone testing and validation during peri-operative anesthesia. A full description is described in our previous work [23]. In brief, this controller is based on a PD model represented by a patient-individualized sigmoid E_max_ model, describing the relationship between BIS and CePROP. The sigmoid E_max_ model can be described by: (1) E_0_: the BIS value at no drug effect; (2) the change in BIS between no drug effect and maximum drug effect is defined as E_max_;3 (EC_50_: the CePROP at 50% of effect; (4) γ: the steepness of drug effect around 50%. The controller estimates the target CePROP that minimizes the error between measured and target value for the controlled variable (BIS) by shifting the sigmoid E_max_ model along the CePROP axis. We improved the model estimator by implementing a Bayesian technique to continuously calculate a patient-individualized sigmoid E_max_ combining an initial population mean model with the observed responses. The Bayesian objective function is:

2 $$\sum {\frac{{{{\left( {BI{S_{sample}} - BI{S_{estimated}}} \right)}^2} \times MAX\left[ {\left( {1 - {{\left[ {\frac{{(t - {t_{sample}})}}{{sampl{e_{TO}}}}} \right]}^2}} \right);\,0} \right]}}{{VAR_{{samples}}^{2}}}} +\frac{{{{\left( {E{C_{50,Population}} - E{C_{50,Estimated}}} \right)}^2}}}{{VAR_{{E{C_{50}}}}^{2}}}+\frac{{{{\left( {{\gamma _{Population}} - {\gamma _{Estimated}}} \right)}^2}}}{{VAR_{\gamma }^{2}}}+\frac{{{{\left( {{D_{Population}} - {D_{Estimated}}} \right)}^2}}}{{VAR_{D}^{2}}}$$

whereby VAR denotes the variance, BIS_sample_ is the observed value and BIS_estimated_ is the estimated value based on the model to be fitted. EC_50_ and γ are the non-fixed terms of the sigmoid Emax model, “Population” is the original population reference model parameter (= *a priori* information), and “Estimated” is the estimated value of parameter for the individual. Sample TO is a forgetting factor representing the samples taken into account for the modelling on a time-limited base, and D is the systems delay to be estimated [24, 26].

**Fig. 1:**
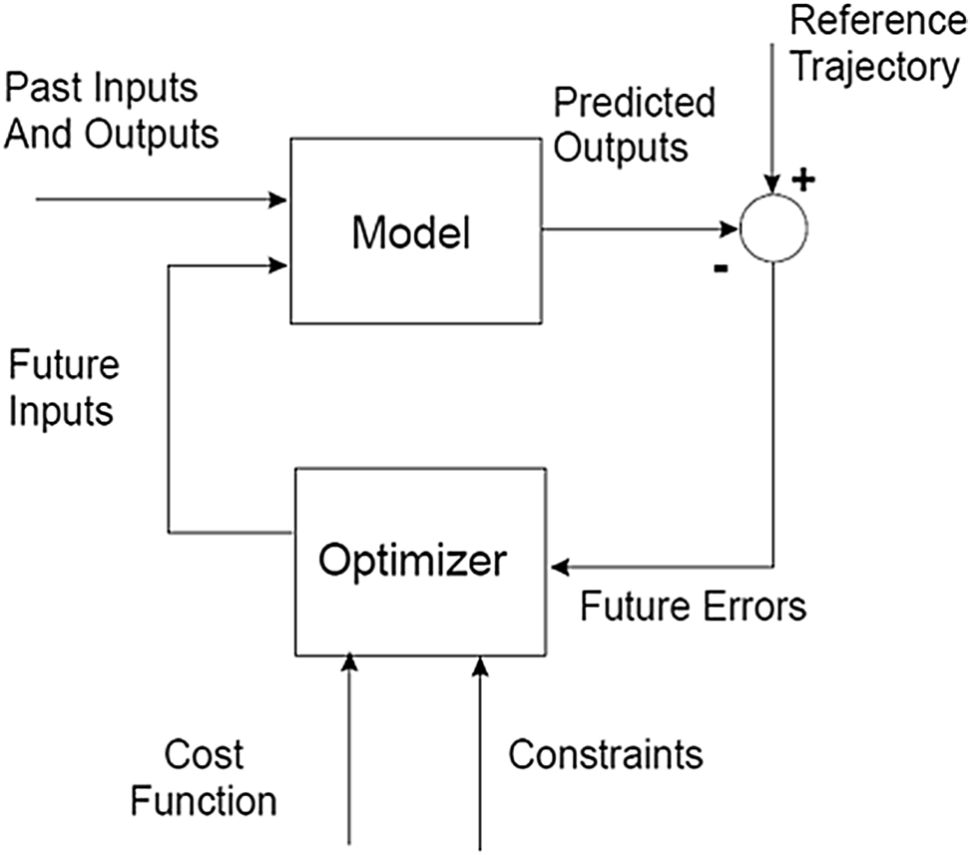
MPC-EPSAC strategy as a block scheme (*MPC* model predictive control, *EPSAC* extended prediction self adaptive control)

For the model based predictive SISO controller (called EPSAC—extended prediction self adaptive control) an algorithm, which had undergone simulator testing, has been deployed in the RUGLOOP II framework [20]. This predictive control algorithm has been described previously [19, 21, 37]. It predicts the future BIS response based on past propofol input rates and past measured BIS values. At every sampled instant t the controller calculates the optimal dose for Propofol over a predefined number of moments in the future (defined by N2 variable), and computes a number of control moves to achieve/to maintain the desired BIS target (defined by Nu variable). Figure 1 represents the concept.

The prediction of future responses in BIS implies minimizing a quadratic cost function representing the error between the target BIS value and the predicted BIS value (denoted BIS sample) at every instant time t, possibly with an additional term penalizing the control effort denoted by variable u:

3 $$\mathop \sum \limits_{{k=N1}}^{{N2}} {\left( {BI{S_{target}}\left( {t+k|t} \right) - BI{S_{sample}}\left( {t+k|t} \right)} \right)^2}+\alpha \mathop \sum \limits_{{k=0}}^{{N2 - 1}} {\left( {u(t+k|t)} \right)^2}$$

where the notation (t + k|t) denotes predicted values over k samples in the future postulated at time instant t, N2 is the prediction horizon in samples, N1…N2 is the coincidence horizon in samples (with default value of N1 = system time delay + 1 sample for causality) and α a weighting parameter for control effort u which denotes the optimized values of propofol infusion rates.

The EPSAC controller design parameters are fixed for all patients and chosen as: *N*_u_ = 1, *N*_1_ = 1 and *N*_2_ = 10. Based on our vast expertise in applying EPSAC, *N*_u_ = 1 is the most simple choice from practical engineering point of view, giving satisfactory performance for stable processes. In the cost function (3), the weighting parameter on the control effort term was not used (i.e. α = 0).

For the “human” SISO controller (called *HUMAN*) the clinician titrated the propofol using an effect-compartmental controlled TCI system to target a BIS between 40 and 60 based on his/her clinical expertise. CeREMI was guided in order to maintain a mean arterial blood pressure (MAP) between 65 and 85 mmHg and to maintain an adequate heart rhythm, depending on the patient’s medical history.

In order to guarantee safety, additional algorithms are incorporated into the closed-loop system in all applied SISO systems. For example, the maximum allowed CePROP is set at 15 µg/ml. When the incoming BIS values are corrupted by noise making closed-loop control unavailable, the BIS signal quality index (SQI) below 50% is used to automatically “open” the loop, continuing the propofol infusion at the most recent Ce_PROP_ and the nurse is alarmed. Closed-loop control remains active and the system will “close” the loop again when accurate BIS levels are available again. Hereby, “control time” is defined as the percentage of the case time the closed-loop control is active. In case of output oscillations in computer-based titration from the automated SISO systems, an auditive alarm sounded and the nurse could take over control of the drug administration in case of emergency, which was then accounted for as a system failure.

### Performance evaluation indicators and statistics

To assess the detailed differences between the controllers over time, the difference between the mean BIS, CePROP and CeREMI, hemodynamic and respiratory values [heart rate (HR), MAP, oxygen saturation (Sp0_2_) and end-tidal CO_2_ (EtCO_2_)] were plotted against time [38]. Hereby, the mean value is the population mean at every time point. The closer the differences between the means were to zero the less difference there was between the means of the variable values of the two controllers. The 95% confidence intervals (CI) for the differences between the mean values were calculated at each 30 s time point. When zero is included in the 95% CI, there is no statistically significant difference.

BIS was defined as the controlled variable. The percentage of the total observed time over which BIS remained between 40 and 60, was calculated for each case. The global performance of the three closed-loop systems can be evaluated using prediction error (PE) and its derived median (absolute) prediction error [MD(A)PE], Wobble and Divergence, as described in Appendix 1, and has been used previously during evaluation of intra-operatively used closed-loop systems [26].

For data with a normal distribution the student *t* test was employed and without normal distribution, we have applied the Mann–Whitney *U* test. Statistical significance was set at 5% and a Bonferroni correction was used to account for multiple comparisons. The corresponding p value of the tests is provided in the results. The software package R (R Foundation for Statistical Computing, Vienna, Austria) was used to perform the statistical analysis.

To investigate the dynamic variability of the system and the required “workload” of the closed-loop system to maintain a specific target, spectrogram analysis and its derived power spectrum density (PSD) has been used as described in Appendix 2.

As this was an observational, pilot-study, no formal power analysis was done and numbers of patients were arbitrary set.

## Results

Thirty-six patients were approached and agreed to participate in the study. In the EPSAC group, 16 patients were included. Two patients were excluded post-hoc due to a technical problem with the data recording. In each of the BAYES and the HUMAN group, 10 patients were included. The patient characteristics were similar between groups (Table 1). The total control time (percentage of case time closed-loop control was active) in closed loop was 93 ± 5.2% and 99 ± 0.7% in the EPSAC and BAYES groups, respectively, and was not statistically significantly different (NS). The total case time was 339 ± 65 min, 361 ± 17 min and 370 ± 6 min for EPSAC, BAYES and HUMAN, respectively (NS).

**Table 1:** Patient characteristics, APACHE II and Euro Score [mean ± SD or median (range)]

	EPSAC	BAYES	HUMAN
Number	14	10	10
Gender (M/F)	12/2	8/2	9/1
Age (year)	66 ± 9	62 ± 7	65 ± 7
Weight (kg)	80 ± 13	80 ± 13	87 ± 13
Height (cm)	174 ± 10	171 ± 8	172 ± 10
APACHE II score	14 ± 4	13 ± 2	12 ± 4
Euro score	2 (0–7)	4 (1–7)	4(0–9)

**Fig. 2:**
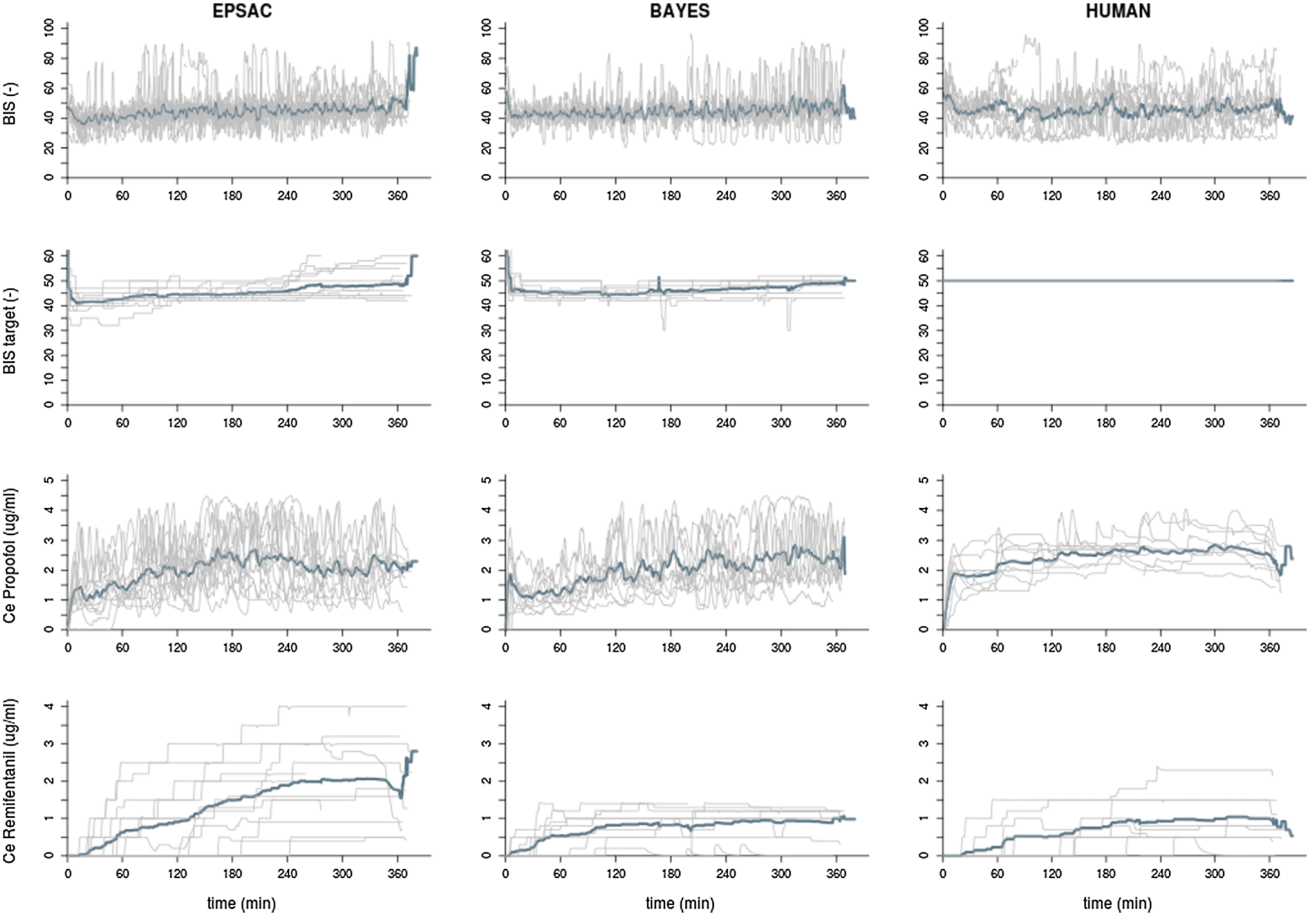
Time course for measured BIS, targeted BIS, predicted propofol effect-site concentration (CePROP), and predicted remifentanil effect-site concentration (CeREMI) for the three groups. Blue line represents population mean value at every time point; grey lines are the data for each individual

**Fig. 3:**
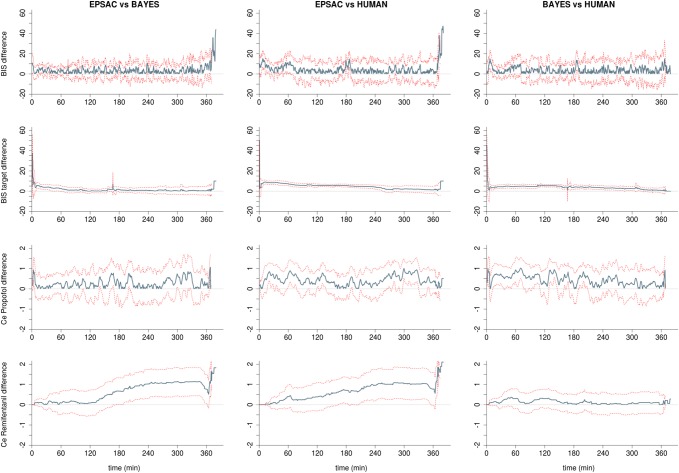
Time-synchronized analysis of the differences between groups for measured BIS, targeted BIS, predicted propofol effect-site concentration (CePROP), and predicted remifentanil effect-site concentration (CeREMI). Blue line represents the absolute difference of the means of both populations at every time point; dotted red lines represent upper and lower 95% confidence interval at every time point

The time course of targeted and measured BIS is shown in Fig. 2. To enable comparison, target BIS for HUMAN was set as 50 being the nadir of the set target range between 40 and 60. The time course of the PE for each controller is also shown in Fig. 2. The targeted and measured median BIS values are shown in Table 2. Similar results for BIS were observed between groups. Figure 3 depicts the detailed differences between controllers for BIS over time. Although some lower targets were set for EPSAC and BAYES than HUMAN, no differences in the time course of measured BIS values were observed between controllers.

**Table 2:** BIS levels, drug usage, hemodynamics and respiratory parameters. Median (range)

	EPSAC	BAYES	HUMAN
Number	14	10	10
BIS	42 (39–49)	43 (41–46)	43 (30–54)
BIS target	44 (42–50)	45 (43–50)	NA
CpPROP (µg/ml)	1.7 (0.9–3.0)*	1.7 (1.0–2.8)**	2.6 (1.9–3.0)
CePROP (µg/ml)	1.8 (1.0–3.1)*	1.8 (1.1–2.7)**	2.6 (1.9–3.0)
CpREMI (ng/ml)	1.1 (0–3.0)	0.9 (0–1.3)	0.6 (0–1.5)
CeREMI (ng/ml)	1.1 (0–3.0)	0.9 (0–1.3)	0.6 (0–1.5)
CtREMI (ng/ml)	1.1 (0–3.0)	0.9 (0–1.3)	0.6 (0–1.5)
MAP (mmHg)	76 (70–95)*^,^***	71 (63–80)	73 (61–81)
HR (beats/min)	78 (60–116)	77 (65–97)	78 (64–92)
EtCO_2_ (kPa)	4.1 (2.8–5.0)	4.2 (3.4–5.3)	4.0 (3.3–4.7)
SpO_2_ (%)	99 (95–100)	99 (98–100)	99 (98–100)

*CpPROP* propofol estimated plasma concentration, *CePROP* propofol estimated effect-site concentration, *CpREMI* remifentanil estimated plasma concentration, *CeREMI* remifentanil estimated effect-site concentration, *CtREMI* remifentanil target concentration. *MAP* mean arterial blood pressure, *HR* heart rate, *EtCO*_2_ end-tidal CO_2_, *SpO*_2_ oxygen saturation

*p < 0.017 between EPSAC and HUMAN control

**p < 0.017 between BAYES and HUMAN control

***p < 0.017 between BAYES and EPSAC control

Overall CePROP and CeREMI are shown in Table 2 and the time courses of CePROP and CeREMI are plotted in Fig. 2. A higher median CePROP was required in the HUMAN group compared the BAYES and EPSAC, also observed in some temporary differences between groups in the course analyses of CePROP (Fig. 3). Although median CeREMI seemed similar among groups, time course analysis of CeREMI (Figs. 2, 3) revealed a significant increase in CeREMI in the EPSAC group compared to BAYES and HUMAN during the case, resulting in significantly higher CeREMI for EPSAC after some hours of sedation.

**Fig. 4:**
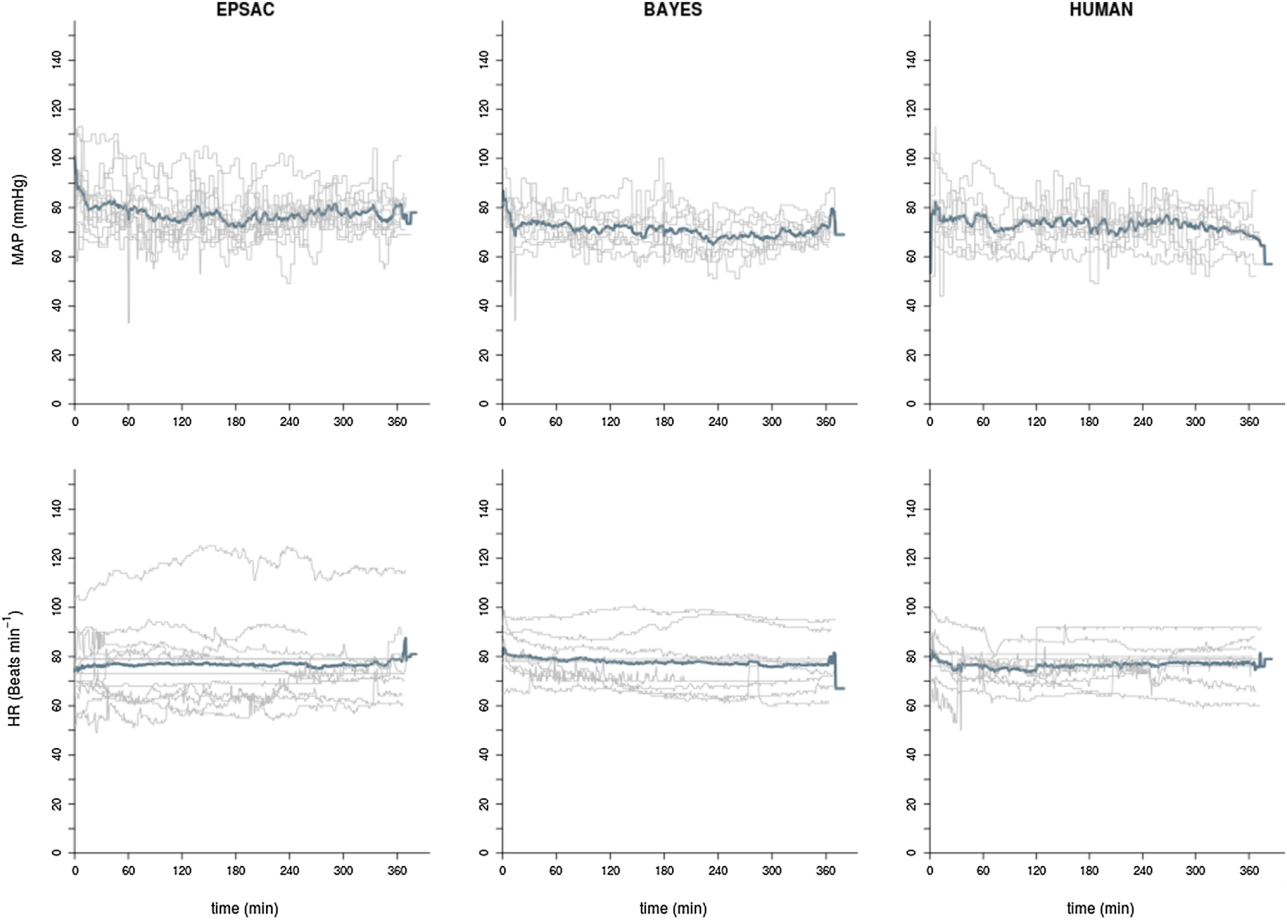
Time course of non-invasive mean arterial blood pressure (MAP) and heart rate (HR) for the three groups. Blue line represents population mean value at every time point; grey lines are the data for each individual

**Fig. 5:**
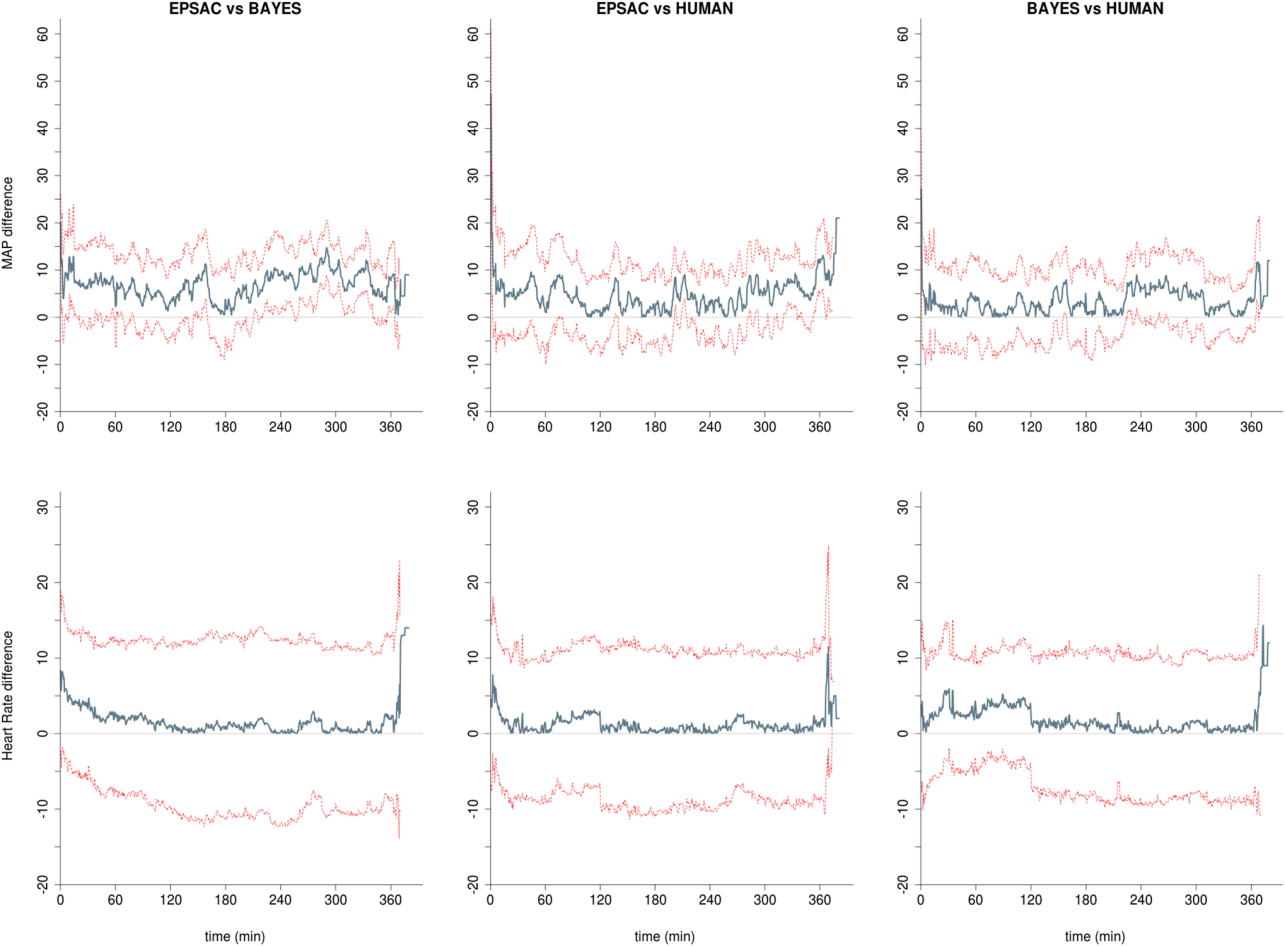
Time-synchronized analysis of the differences between groups for heart rate (HR) and non-invasive mean arterial blood pressure (NIBP). Blue line represents the absolute difference of the means of both populations at every time point; dotted red lines represent upper and lower 95% confidence interval at every time point

Hemodynamic parameters were stable in the three groups, with a statistically, albeit not-clinically relevant, higher levels of MAP for the EPSAC group (Table 2; Figs. 4, 5). As expected during mechanical ventilation, parameters for ventilation (Table 2) were similar among groups.

**Fig. 6:**
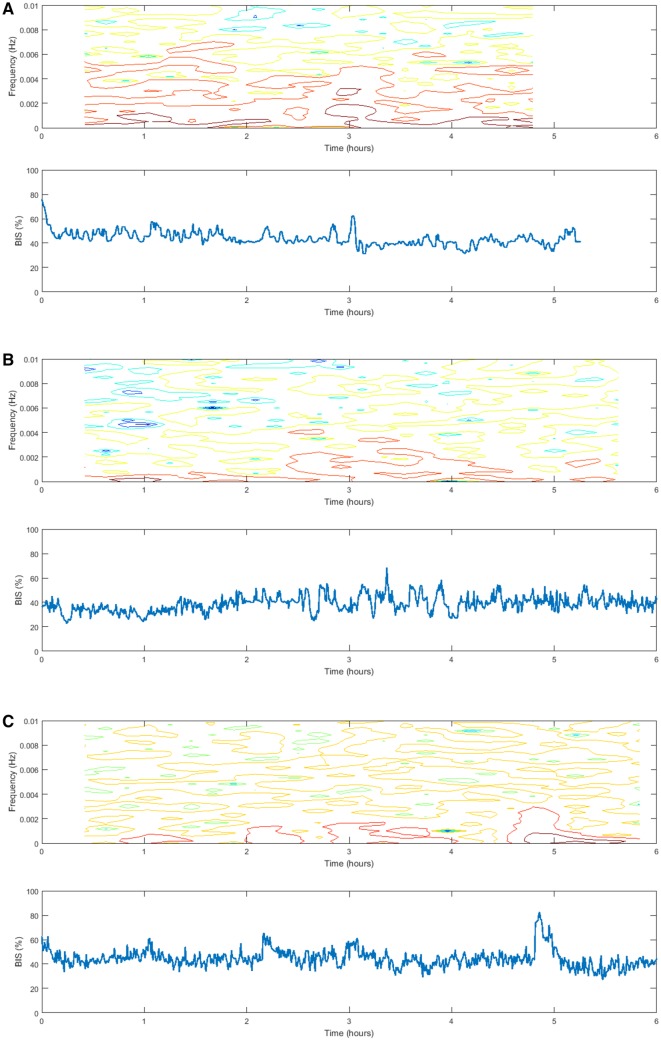
Spectrogram and time signal for measured BIS values in open loop (left) versus closed loop. **a**: BAYES, **b**: EPSAC and **c**: HUMAN

The values for performance indicators are given in Table 3. Similar results between controllers were seen for MDPE and Wobble, however, the HUMAN controller showed a significantly larger MDAPE. Divergence was negative in HUMAN and positive for EPSAC and BAYES. Percentage of case time of BIS with/without certain value ranges is given in Table 4. Percentage of time with accurate BIS control between 40 and 60 was similar between groups due to wide range of results, however, BAYES showed a trend towards better control. The results for the spectrogram and time signal for measured BIS in the three groups is shown in Fig. 6a–c. The result [median (min–max)] for the total PSD were 0.039 (0.0013–0.161), 0.037 (0.007–0.12), and 0.053 (0.0042–0.198) for EPSAC, BAYES and Human, respectively. PSD in HUMAN controlled patients was higher than EPSAC controlled patients (p < 0.008) and the BAYES controlled patients.

**Table 3:** Prediction error [median (minimum − maximum value)]

	EPSAC	BAYES	HUMAN
Median MDPE %	− 4.2 (− 8.2 to − 0.5)	− 6.7 (− 9.3 to − 4.4)	− 14 (− 40.8 to 7.1)
Median MDAPE %	8.4 (4.9 to 15.4)*	9.4 (6.7 to 15)**	16 (7.4 to 41.2)
Median wobble %	7.3 (3.7 to 14.8)	6.2 (5 to 14.4)	9 (5.8 to 20.8)
Median divergence %	0.00325 (− 0.00998 to 0.04043)	0.00839 (− 0.00273 to 0.01709)	− 0.005 (− 0.04742 to 0.07776)

*MDPE* median prediction error for the individual patient, *MDAPE* median absolute prediction error for the individual patient

*p < 0.017 between EPSAC and HUMAN control

**p < 0.017 between BAYES and HUMAN control

**Table 4:** Percentage (%) [median (minimum − maximum)] of case time for specific BIS ranges

	EPSAC	BAYES	HUMAN
% of casetime BIS < 40	27.9 (6.3–56)	21.3 (5.5–45.2)	19.9 (0–8.3)
% of casetime 40 < BIS < 60	67.8 (42.7–90.5)	75 (41.2–92.8)	68.4 (13.9–93.7)
% of casetime BIS > 60	4.1 (0–19.9)	3.7 (0–13.6)	6.9 (2.5–33.2)

## Discussion

This observational study is an evaluation of the performance of three different control strategies for propofol-to-BIS post-operative intensive care sedation. The experimental results indicate that both computer-based control systems are feasible and robust to be used during ICU sedation with overall tighter control than HUMAN and even with lower required CePROP. EPSAC control required higher CeREMI than BAYES or HUMAN to maintain stable control.

BIS was used as the controlled variable in all groups and measured BIS values were similar between groups and represent adequate care in all patients. The period of adequate sedation (% time BIS between 40 and 60), seen as indicator of performance of the automated system was similar among groups, although trended to be higher in BAYES. Interestingly, although BIS time courses are similar between groups, significantly higher CePROP was required to maintain accurate control when using the HUMAN control strategy even with a similar CeREMI with BAYES. Eventhough similar overall CeREMI were co-administered for all control strategies, studying detailed differences with the time-based analysis revealed some higher CeREMI when using EPSAC in the last 3 hours of control to enable accurate control. As higher CeREMI might make the controller task less challenging due to less incoming arousability triggering the BIS to increase, the need for higher CeREMI to stabilize control might indicate lower robustness of the EPSAC system. Additionally, changes in CeREMI will dynamically change the patient’s PD model for propofol in BAYES which favors robustness.

Hemodynamic alterations postoperative in these patients asks for specific care. However, hemodynamic results reflected by the MAP and HR were greatly as good for EPSAC, BAYES and HUMAN. This hemodynamic stability in EPSAC and BAYES was created without changing levels of propofol, also indirectly proving the performance of these closed-loop systems. Results for EtCO_2_ and SpO_2_ are similar as can be expected when mechanical ventilation is used.

MDPE represents the direction (over- or under-control) of the performance errors rather than their size. A negative MDPE value indicates the controller tends to overdose, leading to BIS levels below target, a positive MDPE shows the tendency of a too light anaesthesia. Although no significant differences in average MDPE are observed between controllers, HUMAN shows a very wide range of results for individual MDPE, indicating significant individual variability in bias during control. MDAPE represents inaccuracy of control or ‘the error of the system’ and is a necessary accompanying measure to MDPE which does not indicate the sign of a possible bias but describes both the amplitude of possible bias, as well as all other errors that prevent the controller from approaching the target. MDAPE was significantly different between both controllers, with the manual control showing overall larger inaccuracy than both EPSAC and BAYES controller. The results are comparable as for MDPE, with the worst results for HUMAN. Wobble is an indicator for intra-individual variability. Comparing the three groups and although not significant, BAYES has the lowest value, or the best result, probably indicating the integrating of the adaption inherent in the system to recent individual data of the patient. This result is the best also compared to the wobble obtained with other systems, previously published [30]. Divergence is obtained from linear regression of the absolute PE versus time. It indicates if positive, a progressive widening of the gap between BIS_target_ and BIS_measured_. For all groups, divergence is very low and not significantly different.

Spectrographic analysis of the system behavior [39] shows that a larger spectrum of frequencies are covered in BAYES and EPSAC than HUMAN, being an indication that the computer-controlled closed loops uses a broader spectrum of possible inputs to obtain the optimal solution. Figures of PSD of BIS signal suggest that during time of operation, the variability of the BIS changes its main energy content (changes in color intensity). This can originate from patient artefacts, dynamic response variability, and a broader spectrum of input profiles (propofol) in closed loop compared to open. A greater versatility can be recognized in BAYES and EPSAC when compared to human, necessary since the automatic controller only “sees” the BIS signal, whereas the HUMAN has more accurate information on the patient state. The energy of BIS for the manually controlled patients is higher than closed loop, shown by the analysis of the Total PSD index derived from the BIS spectrograms, implying that the BAYES has the smoothest distribution of energy (i.e. frequency components), thus a more ‘patient-friendly’ approach. The justification is in the continuous adaption of the PD model when using BAYES, opposed to EPSAC or MANUAL.

However, the current study shows also shortcomings. This is a first-use and feasibility study and some of the non-significances might be due to the small number of included patients per group. Nevertheless, significances in propofol use, MDAPE and PSD shows acceptable feasibility of both closed-loop systems. Additionally, the closed-loop controllers are not tuned here for cases when a priori known disturbances are included. For instance, if the surgical manoeuvres are known/predefined, a typical surgical stimulation signal could be constructed, fed to the controller and the optimal rates of propofol calculated with this additional information at hand. This is possible in the Bayesian Controller as a feedforward control scheme, and in the EPSAC controller as a dedicated disturbance filter tuning parameter (additional degree of freedom in optimization process). Information on the feedforward control type of schemes have been extensively described elsewhere [40]. Further studies and bench testing are necessary before possible implementation.

## Conclusions

This observational, open-label, pilot study supports the claim that automatic guided sedation with both EPSAC and BAYES are feasible and robust to be used during post-operative ICU sedation with overall tighter control than HUMAN and even with lower required CePROP. EPSAC control required higher CeREMI than BAYES or HUMAN to maintain stable control.

## Electronic supplementary material

Below is the link to the electronic supplementary material.

Supplementary material 1 (DOCX 52 KB)
