# Supplementary material for: A comparison of propofol-to-BIS post-operative intensive care sedation by means of target controlled infusion, Bayesian-based and predictive control methods: an observational, open-label pilot study
Source: J Clin Monit Comput. 2018 Oct 11;33(4):675–86. doi: 10.1007/s10877-018-0208-2 (PMC6602998; doi:10.1007/s10877-018-0208-2)
Supplement: Supplementary file 1 — Supplementary material 1 (DOCX 52 KB) [file 10877_2018_208_MOESM1_ESM.docx]

Appendix 1 : The Varvel criteria :

| $PE=\frac{BIS_{\text{measured}}-BIS_{\text{target}}}{BIS_{\text{target}}}\times100$ | (4) |
| --- | --- |

with $BIS_{\text{target}}$the value of BIS set by the nurse, who decided to an adequate sedation level. The PE value was calculated according to each $BIS_{\text{target}}$ defined for each patient.

Median performance error (MDPE), median absolute performance (MDAPE) inaccuracy and wobble were calculated. MDPE is a measure of bias describing whether the measured values are systematically either above or below the target value and indicates controller bias without revealing any information on dynamic or higher-frequency behavior or on the amplitude of possible oscillations in control, calculated as :

*MDPE_i_ = median PE_ij_, j=1,…,N_i_* (5)

where *N_i_* is the number of PE values obtained for the *i*^th^ subject.

*Median absolute performance error* (MDAPE) reflects the inaccuracy of the control method in the *i*^th^ subject:

*MDAPE_i_ = median |PE_ij_|, j=1,…,N_i_* (6)

where *N_i_* is the number of values *|PE|* obtained for the *i*^th^ subject.

*Divergence* describes the possible time-related trend of BIS_measured_ in relationship to BIS_target_ or the slope of the linear regression equation of ⎜*PE* ⎜against time, expressed in units of percentage divergence per minute. A positive value indicates progressive widening of the gap between BIS_target_ and BIS_measured_, whereas a negative reveals narrowing of the gap.

*Wobble* is another index of the time-related changes in performance measuring the intra-subject variability in performance errors. In the *i*^th^ subject the percentage of wobble is calculated as follows:

Wobble_i_ = median |PE_ij_ - MDPE_i_|, *j=1,…,N_i_ (7)*

Appendix 2 : Spectrogram calculations :

When a signal is recorded from a sensor, it receives a time stamp. The time stamp denotes the interval at which the signal is recorded. In computer based systems, these recorded signals are called digital signals, where the values is recorded at time instant t, followed by a non-recording interval of period Ts (i.e. the sampling period), and the next recorded value of the signal is at time t+Ts. There is a relationship between a time based digital signal and frequency, through the sampling period Ts. The corresponding frequency domain representation of the digital signal is then represented in the corresponding frequency plane from 0 to half the sampling frequency, i.e. 1/(2*Ts) in units of Hz. The number of frequency domain values in this interval depends on the number of time based signal recordings. The longer we measure – the more number of recorded values – the more fine grid we have in frequency domain of corresponding values.

However, it is possible to characterize a signal in both time and frequency domain. Time-Frequency analysis is one of the approaches that give a wider view towards the signal, using spectrograms, wavelet analysis, short time Fourier transform, etc.

According to Fourier’s theorem, it is assumed that the signal $\text{y }=\text{ }(y_{0},\text{ }y_{1},\text{ }.\text{ }.\text{ }.\text{ },\text{ }y_{N-1})\text{ }$, with *N* the number of samples recorded in the signal, is defined by a sum of sinusoids – this is generic signal processing concept theory. The aim of a time-frequency transform is to discover which sinusoid components are present in the signal, and which are not. Further on, the time-frequency transform will separate the most significant (i.e. the higher power) frequency components in the original time based signal. This is very helpful to eliminate signal recording noise, or other collateral influences from other sources (e.g. 50Hz noise, specific electric/electronic noise, etc).

The short time Fourier transform (STFT) is a method for analysing a digital signal based on the discrete cosine transform (DCT):

| $Y_{m}^{DCT}=c_{m}\sum_{n=0}^{N-1} y_{n}\cos\left(\frac{(2n+1)m\pi}{2N}\right),\text{ }m=0,1,...,N-1$ | (8) |
| --- | --- |

with a scaling factor :

| $c_{m}=\left\{\begin{matrix} \sqrt{1/N};\text{ }m=0 \\ \sqrt{2/N};\text{ }m>0 \end{matrix}\right.$ | (9) |
| --- | --- |

The short-time DCT as:

| $Y_{m}^{DCT}=c_{m}\sum_{k=0}^{\infty} y_{n}w_{k-nN}\cos\left(\frac{(2n+1)m\pi}{2N}\right),\text{ }m=0,1,...,N-1;\text{ }n=0,1,...\infty$ | (10) |
| --- | --- |

with $w_{k-nN}$the window that covers the time domain from *nNT_s_* to (*n*+*1*)*NT_s_* ,selects the sequence in the range $\left[y_{nNT_{s}}...y_{(n+1)NT_{s}}\right)$, with *T_s_* the sampling time. It follows that the STFT is given by:

| $Y_{m}^{DFT}=c_{m}\sum_{k=0}^{\infty} y_{n}w_{k-nN}e^{\frac{j2\pi nm}{ N}},\text{ }m=0,1,...,N-1;\text{ }n=0,1,...\infty$ | (11) |
| --- | --- |

Creating a spectrogram using the STFT is usually a [digital](http://en.wikipedia.org/wiki/Digital_(signal)) process. The [sampled](http://en.wikipedia.org/wiki/Sample_(signal)) signal (i.e. recorded values of our time based signals), in the time domain, is divided into intervals (also referred to as “windows”) of a certain number of time samples $w_{k-nN}$. These windows are usually overlapping, as to analyse in detail any possible transition of the energy in the signal from its various components, and Fourier transformed to calculate the magnitude of the frequency spectrum for each window. Each window then corresponds to a vertical line in the image; a measurement of magnitude versus frequency for a specific moment in time. These are called spectrums or time plots which are then "put side by side" to form the (Jet-coloured) image or a three-dimensional surface. In other words, a spectrogram returns the power spectral density (PSD) of each window, and the units are decibels (dB).

In the case of our signal processing, the overlap between windows was 50%, with Kaiser-filtered windows of length 512 samples and weighting factor of$\beta=5$ (increasing this factor increases the noise attenuation) (reference: Oppenheim, Alan V., Ronald W. Schafer, and John R. Buck. Discrete-Time Signal Processing. Upper Saddle River, NJ: Prentice Hall, 1999, p. 474.). The Kaiser filter is defined by

$$w\left( n \right)=\frac{I_{0}\left( \beta\sqrt{1-\frac{n-\frac{N}{2}}{\frac{N}{2}}} \right)^{2}}{I_{0}(\beta)}$$

where n is the sample in the interval, N the total number of samples in the window interval, I0 is the zeroth-order modified Bessel function of the first kind. This filter is used to provide a smooth convergence of the PSD towards its tails before it is Fourier transformed – which further ensures a good spectrogram resolution.

In general, the spectrogram is an interesting tool for non-stationary signals, such as intra-patient variability and is a plot of these frequency components against time. When applied to the BIS signal, the main components in frequency as denoted by the PSD energy plot, where located up to 0.05Hz – after this frequency value, the signal energy faded at least 5 times and was no longer included in the analysis. For plotting these results, a ‘Jet’ colourmap was used : the more reddish the patch, the greater the magnitude of a certain frequency component over a certain period of time and the less the amplitude, the more deep the blue colour.

To further better understand the performance of the method herein presented, an index was proposed. This index is derived from the spectrogram of BIS signal, called ‘Total PSD’ as the sum of the Power Spectrum Density (PSD) of all frequencies existing in BIS within the frequency interval from 0 Hz to 0.05 Hz, calculated for each time window used to derive the spectrogram. The formula below has been used to calculate such an index after processing the BIS signal, for each patient:

| ${Total PSD}_{ik}=\sum_{f=0}^{0.05} {PSD}_{ik}(f)$ | (12) |
| --- | --- |

where ${Total PSD}_{ik}$ is the Total PSD of the *i*^th^ patient in the *k*^th^ window of the spectrogram and *f* denotes the frequency grid values in units of Hz.

To compare the results among patients in a fair manner, the values of the Total PSD have been normalized between 0-1, where 1 denotes the maximum value from the entire population in each group.
